# Gut bacterial species in late trimester of pregnant sows influence the occurrence of stillborn piglet through pro-inflammation response

**DOI:** 10.3389/fimmu.2022.1101130

**Published:** 2023-01-18

**Authors:** Zhe Chen, Hui Yang, Hao Fu, Lin Wu, Min Liu, Hui Jiang, Qin Liu, Yaxiang Wang, Shuqi Xiong, Mengqing Zhou, Xiao Sun, Congying Chen, Lusheng Huang

**Affiliations:** National Key Laboratory of Pig Genetic Improvement and Production Technology, Jiangxi Agricultural University, Nanchang, China

**Keywords:** gut microbiome, fecal metabolites, stillborn piglet, pro-inflammatory response, sow

## Abstract

Maternal gut microbiota is an important regulator for the metabolism and immunity of the fetus during pregnancy. Recent studies have indicated that maternal intestinal microbiota is closely linked to the development of fetus and infant health. Some bacterial metabolites are considered to be directly involved in immunoregulation of fetus during pregnancy. However, the detailed mechanisms are largely unknown. In this study, we exploited the potential correlation between the gut microbiota of pregnant sows and the occurrence of stillborn piglets by combining the 16S rRNA gene and metagenomic sequencing data, and fecal metabolome in different cohorts. The results showed that several bacterial species from *Bacteroides*, potential pathogens, and LPS-producing bacteria exhibited significantly higher abundances in the gut of sows giving birth to stillborn piglets. Especially, *Bacteroides fragilis* stood out as the key driver in both tested cohorts and showed the most significant association with the occurrence of stillborn piglets in the DN1 cohort. However, several species producing short-chain fatty acids (SCFAs), such as *Prevotella copri*, *Clostridium butyricum* and *Faecalibacterium prausnitzii* were enriched in the gut of normal sows. Functional capacity analysis of gut microbiome revealed that the pathways associated with infectious diseases and immune diseases were enriched in sows giving birth to stillborn piglets. However, energy metabolism had higher abundance in normal sows. Fecal metabolome profiling analysis found that Lysophosphatidylethanolamine and phosphatidylethanolamine which are the main components of cell membrane of Gram-negative bacteria showed significantly higher concentration in stillbirth sows, while SCFAs had higher concentration in normal sows. These metabolites were significantly associated with the stillborn-associated bacterial species including *Bacteroides fragilis*. Lipopolysaccharide (LPS), IL-1β, IL-6, FABP2, and zonulin had higher concentration in the serum of stillbirth sows, indicating increased intestinal permeability and pro-inflammatory response. The results from this study suggested that certain sow gut bacterial species in late trimester of pregnancy, e.g., an excess abundance of *Bacteroides fragilis*, produced high concentration of LPS which induced sow pro-inflammatory response and might cause the death of the relatively weak piglets in a farrow. This study provided novel evidences about the effect of maternal gut microbiota on the fetus development and health.

## Introduction

Litter size is an economically important trait in pig production (1). Over the past decades, the reproduction performance of sows has been greatly improved through genetic improvement (2). However, this also causes an unexpected increase in the number of stillborn piglets. The occurrence of sows giving birth to stillborn piglets varies between 5% to 10%, even as high as 14% in some high prolific herds (3, 4). Some studies have shown that pregnant sows infected with diseases (5), such as sow syndrome endometritis (SSE), porcine pseudorabies (PPR), classical swine fever (CSF) and porcine parvovirus (PPV), could directly result in the occurrences of stillbirth and mummification (6–8). Environmental factors, such as temperature and humidity, also influence the numbers of stillborn piglets in sows (9–11). Sows in the first parity have a higher probability of stillborn piglets, and within certain parities, the probability of stillborn piglets is decreased following the increase of parties (12).

Recently, the studies in humans have indicated that the colonization of microbes in the vagina showed a link with adverse pregnancy outcomes (13, 14). Maternal colonization with *Streptococcus agalactiae* causes neonatal disease and stillbirth (15). Danish et al. reported that *Listeria monocytogenes* in the gut of pregnant mother showed a serious threat to the fetus, even the rate of miscarriage or stillbirth was as high as 32% (16). In mice, intake of high-fat diet could result in placental hypoxia which impaired the development of the fetus (17). *Bifidobacterium* in maternal gut microbiota promotes placental morphogenesis, nutrient transport and fetal growth in mice (18). By far, whether the alteration of gut microbiota causes the occurrence of stillborn piglets in sows has still unknown.

Maternal gut microbiota could drive the early immune development of the offspring by microbial metabolites, such as the authentic ligands for Aryl hydrocarbon receptor (AhR) and short chain fatty acids (SCFAs) translocating to the fetus (19). SCFAs exhibit its functional capacity to increase the number of regulatory T lymphocytes (20). Lipopolysaccharide (LPS), a component of the outer membranes of gram-negative bacteria, could stimulate strong immune responses (21) and plays an important role in increasing the oxidative stress and overproduction of inflammatory cytokines, such as interleukin -1β (IL-1β) and interleukin -6 (IL-6) (22). Dysbiosis of the gut microbiota may increase the production of microbial LPS that activates the inflammatory response and promotes the activation of Toll-like receptor 4 (TLR4) (23–25). Impairment of the intestinal epithelial barrier has been recognized as a crucial factor for the inflammation and immune-mediated disorders (26, 27). Elevated levels of serum zonulin and fatty acid-binding protein 2 (FABP2) have been often recognized as the biomarkers for increased intestinal permeability (28, 29). Hitesh et al. reported that abnormal immune rejection during pregnancy in humans may lead to stillborn fetus, premature birth and other adverse pregnancy phenomena (30). Therefore, we hypothesized that dysbiosis of sow gut microbiota might lead to an abnormal host immune response which caused fetal death and resulted in the occurrence of stillborn piglets.

**Figure 1:**
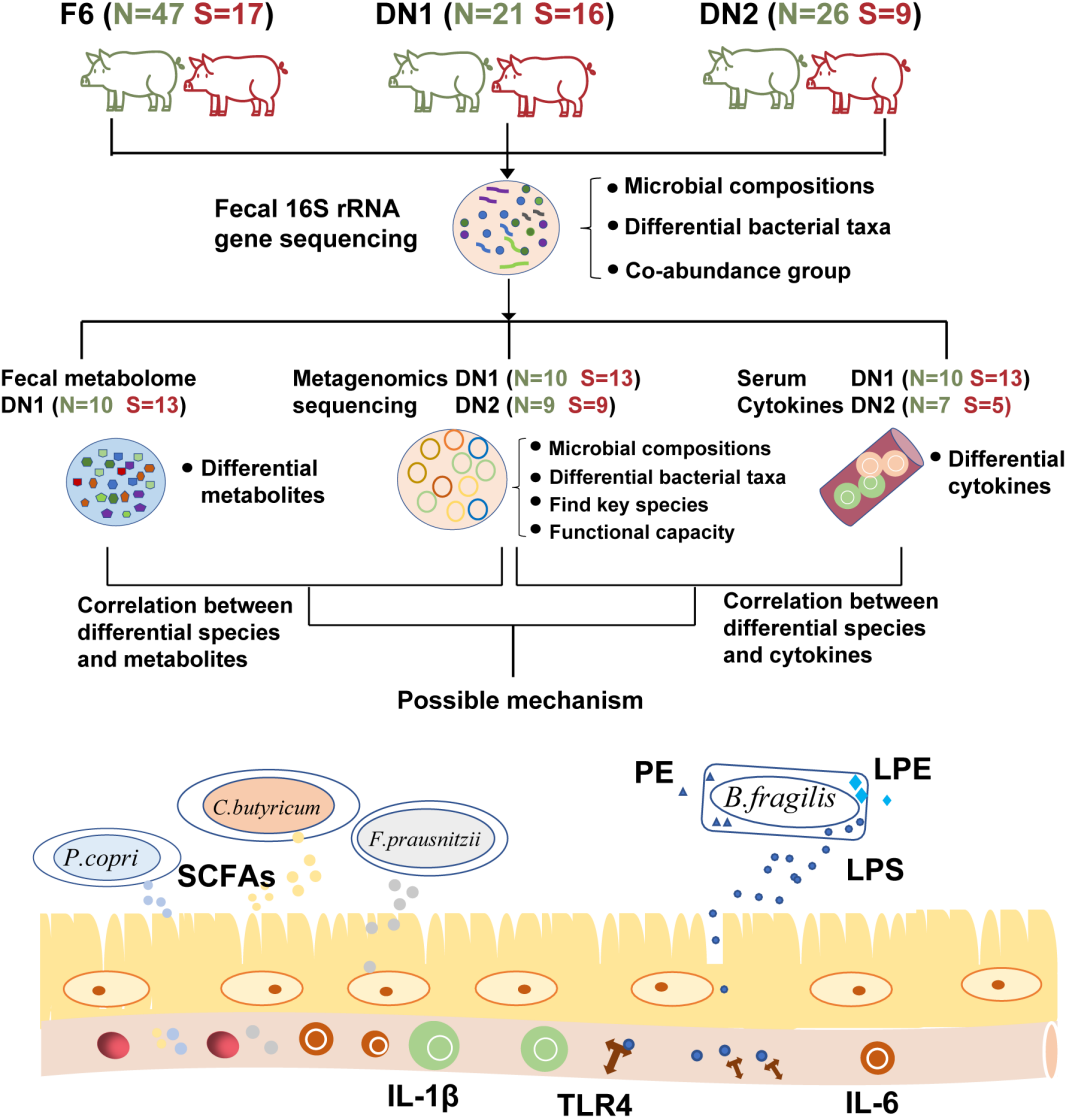
The overall workflow of this study. This plot describes the workflow of this study and shows the category of the multi-omics analysis performed in each cohort. “N” represents Normal group, “S” shows Stillbirth group.

In this study, we performed 16S rRNA gene and metagenomic sequencing analysis to document the association between the occurrence of stillborn piglets and maternal gut microbiota in sows from three cohorts. In addition, we determined the fecal metabolome profiling of pregnant sows using the widely targeted metabolome and lipidomics measurements. Gas chromatograph was used to measure the concentration of fecal SCFAs. We further measured the serum levels of pro-inflammatory cytokines and LPS to assess the immune response of sows. We revealed a potential association between maternal gut microbiota and the occurrence of stillborn piglets in sows by using multiple-omics data, and primarily elucidated the mechanism of maternal gut microbes affecting the occurrence of the stillbirth (**Figure 1**). The results gave a new insight for modulating the gut microbiota of pregnant sows to reduce the occurrence of stillbirth.

## Materials and methods

### Animals, and fecal and serum sample collection

A total of 136 pregnant sows from three experiment cohorts were used in this study. Sixty-four pregnant sows including 17 sows having stillborn piglets (Stillbirth group) and 47 sows giving birth normally (Normal group) were from the 6^th^ (F_6_ cohort) generation of a mosaic population which was constructed through hybridization of four Chinese indigenous pig breeds (Bamaxiang, Erhualian, Laiwu, and Zang) and four commercial breeds (Pietrain, Duroc, Landrace, and Large White). The other 72 pregnant sows were all from a Barkshire × Licha intercross from DingNan farm, including 37 sows raised in the year of 2017 (DN1 cohort, including 16 sows having stillborn piglets, and 21 sows giving birth normally), and 35 sows raised in the year of 2019 (DN2 cohort, including 9 sows having stillborn piglets and 25 normal sows). Pregnant sows were housed in gestation stalls individually before giving birth. And then, all sows were moved from the gestation stalls to the farrowing rooms at the gestation of 100 days. All the sows from the same cohort were provided with the same diets without any antibiotics, probiotics or other medicines (**Supplemental Table S1**). The details about phenotypic records are shown in the **Supplemental Table S2**. The sows with the number of stillborn piglets ≥ 2 in a litter were classified into the stillbirth sow group, while the sows whose all piglets were born alive were classified into the normal group. Fresh fecal samples and serum samples from experimental pregnant sows were collected one week before giving birth. All fecal samples were immediately dipped in liquid nitrogen, and then stored at -80°C until use.

### 16S rRNA gene sequencing and data analysis

Microbial DNA was extracted from 200 mg of each fecal sample with a QIAamp Fast DNA Stool Mini Kit (Qiagen, Germany) according to the manufacturer’s instructions (31). The DNA integrity and concentration were measured with a Nanodrop1000 (Thermo Scientific, USA) and 0.8% agarose gel electrophoresis. The V3 - V4 hypervariable region of the 16S rRNA gene was amplified with the barcode fusion primers (338F:5-ACTCCTACGGGAGGCAGCAG-3, 806R:5-GGACTACHVGGGTWTCTAAT-3). The PCR products were purified with AmpureXP beads (AGENCOURT, USA). After purification, the PCR products were used for library construction and sequenced on an Illumina MiSeq platform (Illumina, USA).

Sequence reads with low Phred score and low quality were removed from raw data to obtain clean reads using QIIME (v1.9.1) pipeline (32, 33). Paired-end sequences from clean reads were assembled into tags using FLASH (v.1.2.11) (34). To avoid the bias of the sequencing depth, we rarefied the sequencing depth to 35,462 sequence reads per sample (the lowest number of sequence reads in tested samples) using the rarefy function in the R package (35). High-quality tags were clustered into operation taxonomic units (OTUs) at the 97% sequence identity with the USEARCH (v7.0.1090) (36). Taxonomy assignments for 16S rRNA gene sequences were performed with the RDP classifier program (V2.2) (37). Principal coordinates analysis (PCoA) was used to document the phylogenetic compositions of gut microbiota. The Analysis of similarities (ANOSIM) was used to compare the gut microbial composition among cohorts. Canonical Correspondence Analysis (CCA) was used to investigate the effects of variates including population, parity and maternal infanticide on the composition of gut microbiota. The α-diversity indices including richness, chao, ACE, Simpson and Shannon index were calculated by R software (v4.2.1) with vegan package (38). A Wilcoxon rank-sum test was used to compare the α-diversity of gut microbiota between stillbirth and normal sow groups (39). We used LEfSe (http://huttenhower.sph.harvard.edu/galaxy/) and Wilcoxon rank-sum test to identify the differential bacterial taxa and KEGG pathways between stillbirth and normal sows. The abundance profiles of bacterial taxa were transformed with a central log-ratio transformation using compositions package in R software (v4.2.1) (40). The 16S rRNA sequencing data was submitted to the CNGB database with the accession number: CRA008769.

### Metagenomic sequencing and bioinformatic analysis

All 72 fecal samples from DN1 and DN2 cohorts were selected for metagenomic sequencing on a Novaseq 6000 platform. DNA libraries were constructed following the manufacturer’s instruction (Illumina, USA). Adaptor and low quality reads were removed from the raw reads by fastp (v0.19.4) (41). BWA was used to filter the reads with high sequence similarity with host genome (42). The clean reads of each sample were assembled by MEGAHIT (v1.1.3) with the option ‘–min-count 2 –k-min 27 –k-max-step 10 –min-contig-len 500’ (43). Non-redundant contigs were generated by clustering all contigs with 100% sequencing identity and 100% coverage using CD-HIT (44). The contigs with length more than 500bp was used to predict open reading frames (ORFs) by the MetaGeneMark software (45). Cd-hit software was used to remove the redundant genes from all predicted genes with the threshold of 95% sequence identity and 90% coverage. Taxonomic profiling was generated by mapping the non-redundant genes into the NCBI-NR database by DIAMOND (v0.9.24) with e-values ≤ 1e−5 (46). For those genes that were matched to multiple distinguishable taxonomic groups (with multiple records of e-values ≤1e−5), the unique taxonomic classification was determined based on the lowest common ancestor algorithms by BASTA (v1.3.2.3) (47) at the thresholds of an alignment length > 25, identity > 80%, and shared by at least 60% of hits. Genes whose encoding proteins could not be mapped to the database were defined as unknown genes. The non-redundant gene catalog was then aligned to the KEGG pathways according to their protein sequences (48). The functional terms of KEGG pathways were determined by KOBAS (v2.0) software (49). After that, the clean reads of each sample were aligned to unique genes in the catalog using BWA MEM(v0.7.17-r1188) (50). The output files were converted to BAM format by Samtools (51). FeatureCounts (V2.0.1) (52) was then used to compute the number of successfully aligned reads. The abundance was normalized to fragments per kilobase of gene sequence per million reads mapped (FPKM) (53). The abundances of microbial taxa, KEGG Orthology (KO), and KEGG pathways were calculated by adding the abundances of all the members falling within each category. Spearman correlation analysis was used to calculate the association between differential bacterial taxa and differential functional pathways. The metagenomic sequencing data was submitted to the CNGB database with the accession number: CRA008770.

### Measurement of fecal short-chain fatty acids by gas chromatograph

A total of 23 fecal samples including 13 samples from sows giving birth to stillborn piglets and 10 samples from normal sows in the DN1 cohort were measured the concentrations of SCFAs including acetic acid, propionic acid, isobutyric acid, butyric acid, isopentanoic acid and pentanoic acid. In brief, about 0.3 g aliquot of fecal sample was mixed with 1,500 ml of DNase/RNase-Free water, homogenized for 30 s, and centrifuged at 5,000 rpm for 4 min. The supernatant was pipetted into a new tube, mixed with 240-μL liquor of metaphosphoric acid and crotonic acid (1:1, v/v), and centrifugated at 15,000 rpm for 15 min. The supernatant was filtered through a 0.22 µm filter membrane (Millipore Express, Germany). Finally, 1,000 µl of filtrate was accurately transferred into a GC vial. Water was used as a blank control to correct the background. Samples were loaded to a GC-gas chromatograph (Shimadzu, Japan) equipped with a flame ionization detection and a thin-film capillary column DB-FFAP (Shimadzu, Japan). LabSolutions software (Shimadzu, Japan) was used for data collection and processing (54).

### Metabolome profiling of fecal samples by a widely targeted metabolome analysis

Metabolome profiles were measured in 23 fecal samples described above from the DN1 cohort (stillbirth group: 13 samples, normal group: 10 samples). Briefly, approximate 50 mg of fecal samples were mixed with 1 ml of water-methanol-acetonitrile (1:2:2). The mixture was vortexed for 1min, homogenized at 45 Hz for 4 min, and incubated at -20°C 2 h. And then, the mixture was centrifuged at 12,000 rpm for 10 min at 4°C. An ultra-performance liquid chromatography coupled with quadrupole time-of-flight mass spectrometry (UPLC-QTOF/MS) was used to detect the metabolites of supernatant. MassLynx software (Waters Corp, USA) was used for data acquisition and system control. Leucine-enkephalin was used as an external standard at a concentration of 100 ng/mL. We used Progenesis QI software (v2.0, Nonlinear Dynamics, UK) to process the preliminary data (55). MetaScope package in Progenesis QI was used to annotate fecal metabolites using the HMDB database based on MS/MS fragmentation data, retention time, neutral mass, isotope distribution and the collisional cross-sectional area (56). The annotation results were processed by removing peaks with missing values in more than 50% of QC samples and 80% of tested samples. The retained peaks were normalized to the QC samples using support vector regression algorithm of “MetNormalizer” in R package (57). A threshold of 30% was set for the relative standard deviation in the QC samples to assess the repeatability of metabolomic data sets (58). We applied Partial Least Squares Discriminant Analysis (PLS-DA) to detect the differential metabolites by MetaboAnalyst 4.0 (59). The metabolites with variable importance in the projection (VIP)>1.5 were selected for further analysis. A Wilcoxon rank-sum test was used to identify the differential metabolites between stillbirth and normal groups, and a false discovery rate (FDR) < 0.05 was set as the significance threshold (39). Spearman correlation analysis was used to test the correlations between gut microbiome and fecal metabolites.

### Comprehensive untargeted lipidomic analysis of fecal samples

The lipidomic metabolites of fecal samples were determined in 23 fecal samples from the DN1 cohort described above. In brief, about 20 mg feces from each sample was thawed on ice, homogenized with 1mL of mixture including methanol, methyl-tert-butyl ether (MTBE) and internal standard substance. Two hundred micro liter of water was added into the mixture, vortexed for 1 min, and then centrifuged at 12,000 rpm at 4°C for 10 min. We extracted 300 μL of the supernatant for measuring the lipidomic metabolites by LC-ESI-MS/MS system (SCIEX, USA). The effluent was connected to an ESI-triple quadrupole-linear ion trap (QTRAP)-MS. LIT and triple quadrupole (QQQ) scans were acquired on a triple quadrupole-linear ion trap mass spectrometer (QTRAP) equipped with an ESI Turbo Ion-Spray interface. The qualitative and quantitative data were generated based on the Metware database (MWDB) created by MetWare Biotechnology Co., Ltd. (Wuhan, China) using secondary mass-spectrometry data. The Analyst software (1.6.3) and MultiaQuant software were used to process the final lipidomic data (60, 61). The Partial Least Squares Discriminant Analysis (PLS-DA) was used to assess the differential lipidomic metabolites by MetaboAnalyst 4.0. The lipidomic metabolites with VIP score >1.5 were selected for further analysis (59). Wilcoxon rank-sum test was used to further identify the significantly differential features between stillbirth and normal group (39).

### Determining the concentration of LPS, biomarkers for intestinal permeability, and proinflammatory cytokines in serum samples

The concentration of serum LPS, FABP2, zonulin, TLR4, IL-1β and IL-6 were determined by commercial enzyme-linked immunosorbent assay (ELISA) kits (ThermoFiser, USA) following the manufacturer’s instructions. Briefly, 50 μL of each diluted serum sample was added into the 96-well microtiter plate coating with the primary antibody, and then, 100 μL of HPR-conjugated secondary antibody was added after incubation for 2.5 h at room temperature. Microtiter plates were washed six times with washing buffer. A total of 50 μL of enzymatic reaction termination solution was added to each sample. A microtiter plate reader (Tecan Infinite 200 pro, Switzerland) was used to measure and record OD value at 450 nm. A standard curve was plotted according to the OD value. Finally, the concentration of LPS, FABP2, zonulin, TLR4, IL-1β and IL-6 in each sample were calculated using the standard curve. Because serum samples were not enough, only LPS, FABP2 and zonulin were measured in the DN1 cohort, while LPS, IL-1β and IL -6 were measured in the DN2 cohort. The Wilcoxon rank-sum test was used to compare the concentration of LPS, FABP2, zonulin, TLR4, IL-1β and IL-6 between stillbirth and normal sow groups. Spearman correlation analysis was used to calculate the association between gut microbes, and proinflammatory cytokines and LPS.

### Construction of co-abundance groups of bacterial taxa

The gut microbiota is a complex micro-ecosystem. Microbial taxa with similar requirements and functions would constitute an ecologic co-abundance group (62). Therefore, a CAG analysis was applied to identify the differential CAGs between stillbirth and normal sow groups based on the abundances of OTUs. Specifically, the OTUs with relative abundance > 0.1% (core OTUs) were selected to construct CAGs with SPIEC-EASI package in R software. In brief, the SparCC correlation coefficient matrix between pair OTUs on the abundance was calculated by the SparCC function of SPIEC-EASI package in R software (63). After that, the correlation coefficient matrix was converted to a distance matrix (1-correlation coefficient), and the Ward’s hierarchical clustering method was applied to cluster OTUs into single CAG based on the distance matrix and permutational multivariate analysis of variance (PERMANOVA) with 999 permutations. The CAGs containing OTUs with correlation coefficient values > 0.5 were displayed in the co-abundance network by Cytoscape v3.7.0 (64). The mean value of relative abundances of the OTUs that were contained in the CAG represented the abundance of that CAG. A Wilcoxon rank-sum test was used to compare the abundances of CAGs between stillbirth sows and normal controls (39). Furthermore, Netshift (https://web.rniapps.net/netshift/) tool was used to identify the key nodes of bacterial species, which might act as an important “driver” in the sub-networks (65).

### Prediction analysis by random forest model

Random forest analysis was used to construct the prediction model to identify the potential biomarkers that could predict the sows giving birth to stillborn piglets using “randomForest” in the R (v4.2.1). A total of 17 differential bacterial species, 36 differential KOs, 56 differential metabolites, and three inflammation cytokines were provided as input variables. We used a nested ten-fold cross-validation to detect the important features. The area under curve (AUC) index and receiver operating characteristic (ROC) analysis were used to evaluate the efficacy of possible cutoff values of the tests with 13 stillbirth sows and 10 normal sows from the DN1 cohort which had both metagenome and metabolome dataset by the pROC package in the R (v4.2.1) (66).

## Results

### Identification and replication of bacterial taxa enriched in the gut of sows giving birth to stillborn piglets in three cohorts based on the 16S rRNA gene sequencing data

**Figure 2:**
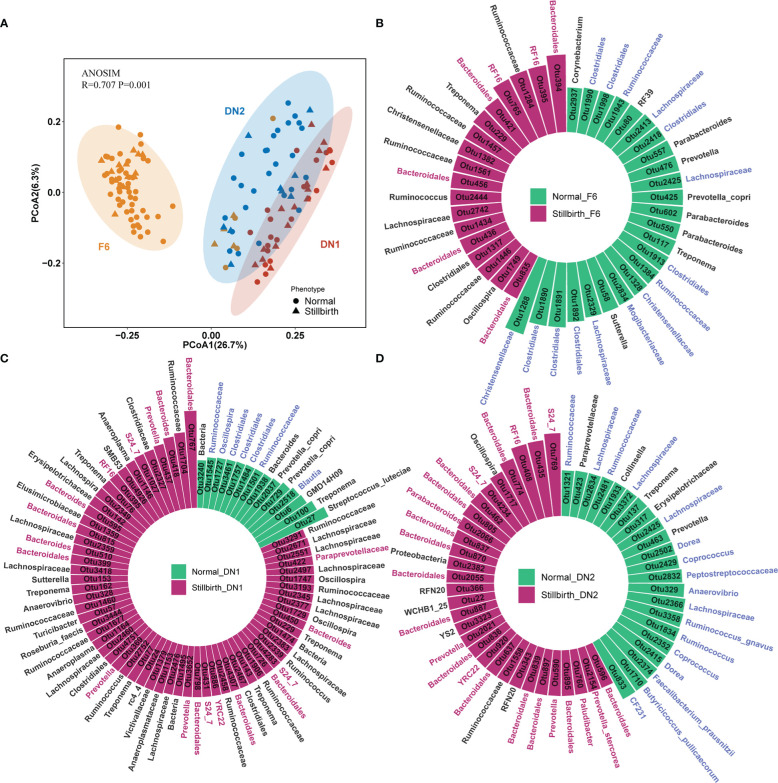
Comparison of bacterial compositions and identification of OTUs associated with sows giving birth to stillborn piglets in three cohorts based on 16S rRNA gene sequencing data. **(A)** PCoA analysis based on the relative abundance of OTUs. ANOSIM was used to compare the gut microbial composition among three cohorts. **(B–D)** The OTUs showing significantly differential abundances between stillbirth and normal sows in the F6 cohort **(B)**, DN1 cohort **(C)** and DN2 cohort **(D)** by LEfSe. The circle histogram showing the OTUs with the LDA score > 2. Blue taxa names represent the OTUs belonging to the order Clostridiales, and red taxa names represent the OTUs belonging to the order Bacteroidales. Green bars indicate the OTUs enriched in the gut of normal sows, and red bars show the OTUs enriched in stillbirth sows.

A total of 136 samples from three cohorts were performed 16S rRNA gene sequencing. After quality control, we obtained 40,555 high-quality tags per sample in average. These tags were clustered into 4,997 OTUs according to the 97% sequence identity. The PCoA plot showed that the compositions of gut microbiota had an obvious difference among three cohorts (**Figure 2A**). Compared to the F6 population, the DN1 cohort showed a higher similarity in the gut microbial composition with the DN2 cohort. This should be due to the fact that two DN cohorts came from the same farm although these sows were raised in different years. CCA analysis was used to evaluate the influence of variates including population, parity, and maternal infanticide on the composition of gut microbiota. Different population had the most significant influence on the gut microbial composition rather than other variates (**Figure S1A**). The phylogenetic composition of gut microbiota had no significant difference between stillbirth and normal sow groups in all three cohorts. Additionally, we compared the α-diversity of gut microbiota between stillbirth group and normal sow group using the chao, richness, ACE, Shannon and Simpson index, and found that the fecal samples from the stillbirth sow group had a higher α-diversity of microbial composition than that from normal sows in all three cohorts although this did not achieve the statistical significance level (**Figures S1B**, **S1C**, **S1D**).

To identify differential bacterial taxa between stillbirth and normal sow group, we first performed a LEfSe analysis with the OTUs having the relative abundance > 0.01% in each cohort. In the F6 cohort, a total of 42 OTUs showing differential abundances between two sow groups were detected, of which 24 OTUs (15/24 OTUs belonging to the order Clostridiales) were enriched in normal sows, and 18 OTUs (7/18 OTUs belonging to the order Bacteroidales) showed enrichment in the gut of sows giving birth to stillborn piglets (**Figure 2B**). In addition, we identified 77 differential OTUs in the DN1 cohort (**Figure 2C**). Among them, 14 OTUs had higher abundance in the normal sow group, half of which were annotated to the order Clostridiales, such as Ruminococcaceae, *Blautia* and *Oscillospira*, while 63 OTUs were enriched in the stillbirth sow group, including 18 OTUs belonging to the order Bacteroidales. In the DN2 cohort, we detected 52 OTUs that had significantly different abundance between stillbirth and normal sow groups (**Figure 2D**). Most of the OTUs enriched in the normal sow group were also annotated to the order Clostridiales (17/22), such as *Dorea*, *Coprococcus*, Ruminococcaceae, *Faecalibacterium prausnitzii*, *Butyricicoccus pullicaecorum*. A total of 30 OTUs had higher abundance in the stillbirth sow group (**Supplementary Table S3**). Among these 30 OTUs, 23 OTUs belonged to the order Bacteroidales.

And then, we used the Wilcoxon rank-sum test to further validate the differential bacterial taxa between stillbirth and normal sow groups with the OTU abundance data through a central log-ratio (CLR) transformation. In the F6 cohort, a total of 62 OTUs showing different abundances between two sow groups were identified. Among them, 23 OTUs (9/23 OTUs belonging to the order Bacteroidales) were enriched in stillbirth sows, while 39 OTUs (26/39 OTUs belonging to the order Clostridiales) had higher abundance in normal sows (**Figure S2A**). A total of 75 differential OTUs were detected in the DN1 cohort, of which 16/43 OTUs belonging to the order Bacteroidales were significantly enriched in the stillbirth group, while 17/32 OTUs annotated to Clostridiales had higher abundance in the normal sow group (**Figure S2B**). In the DN2 cohort, 97 differential OTUs were isolated. Similar to the results obtained in the F6 and DN1 cohorts, most of the OTUs (46/54) enriched in the stillbirth sow group belonged to Bacteroidales, and a large part of OTUs enriched in normal sows were annotated to Clostridiales (**Figure S2C**). As we expected, the high similarity of the association results was obtained between LEfSe analysis and Wilcoxon rank-sum test (**Supplement Table S3**).

**Figure 3:**
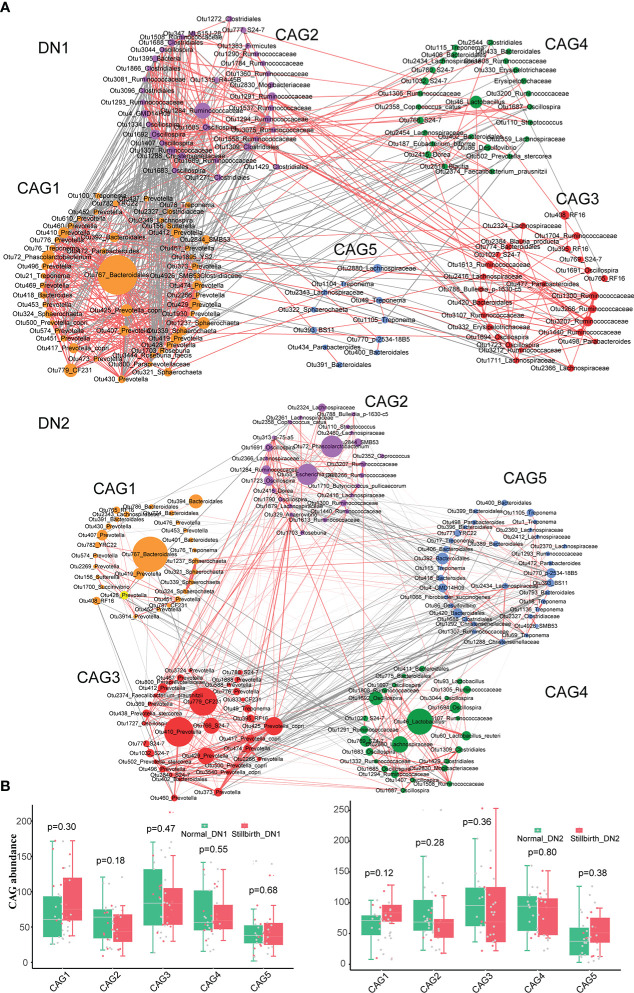
Identifying the co-abundance groups (CAGs) of gut mirobota associated with sows giving birth to stillborn piglets by co-abundance network analysis based on 16S rRNA gene sequencing data. **(A)** Co-abundance networks of DN1 and DN2 cohort. Each circle represents one OTU. Circles with the same color represent the OTUs from CAG. Circle size represents the average abundance of each OTU in CAGs. The red lines represent the positive correlation. And the grey lines show the negative correlation. **(B)** The boxplots showing the differential abundance of CAGs between stillbirth and normal sow group in the DN1 and DN2 by the Wilcoxon rank-sum test.

A co-abundance network analysis was performed in this study to find the CAGs having different abundance between stillbirth and normal sow groups. A total of 156, 182 and 171 core OTUs in the F6, DN1 and DN2 cohort, respectively, were used to construct CAGs by the SparCC correlation and PERMANOVA (**Figures 3A**, **3B**, **S3A**, **S3B**). A total of five CAGs were obtained for each cohort. Those OTUs with correlation coefficient values > 0.5 were displayed in the co-abundance networks. In the DN1 cohort, CAG1 and CAG5 were enriched in the stillbirth sow group, while CAG2, CAG3 and CAG4 had higher abundance in the normal sow group although this did not achieve the statistical significance level. More details, CAG1 was comprised of 50 OTUs. Among these 50 OTUs, 31 OTUs belonged to the order Bacteridales. CAG5 contained 11 OTUs including four OTUs belonging to the order Bacteridales. CAG2 was comprised of 35 OTUs including 21 OTUs annotated to the order Clostridiales. Impressively, CAG1 showed a negative correlation with CAG2 significantly (p=0.001 r=0.12) (**Figures 3A**, **3B**). In the DN2 cohort, CAG1 containing 20 OTUs from the order Bacteridales and CAG5 containing 11 OTUs from the order Bacteridales were enriched in the stillbirth sow group. However, CAG2 was enriched in the normal sow group, in which 21 OTUs were annotated to the order Clostridiales. Interestingly, more potential SCFAs-producing bacterial taxa were enriched in the CAG2, such as OTU72*_Phascolarctobacterium* and OTU1710_*Butyricicoccus pullicaecorum.* (**Figures 3A**, **3B**). Similarly, in the F6 cohort, the OTUs annotated to order Clostridiales mainly constituted CAG1 which exhibited higher abundance in the normal sow group (**Figures S3A**, **S3B**). Taken together, the co-abundance network analysis showed that the CAGs which mainly contained the OTUs belonging to Bacteridales were enriched in the stillbirth sow group, while CAGs composed of OTUs from Clostridiales were more abundant in the normal sow group. This result was consistent with the results from both LEfSe analysis and Wilcoxon rank-sum test.

### Metagenomic sequencing detected bacterial species and functional capacities of gut microbiome enriched in the gut of sows giving birth to stillborn piglets

**Figure 4:**
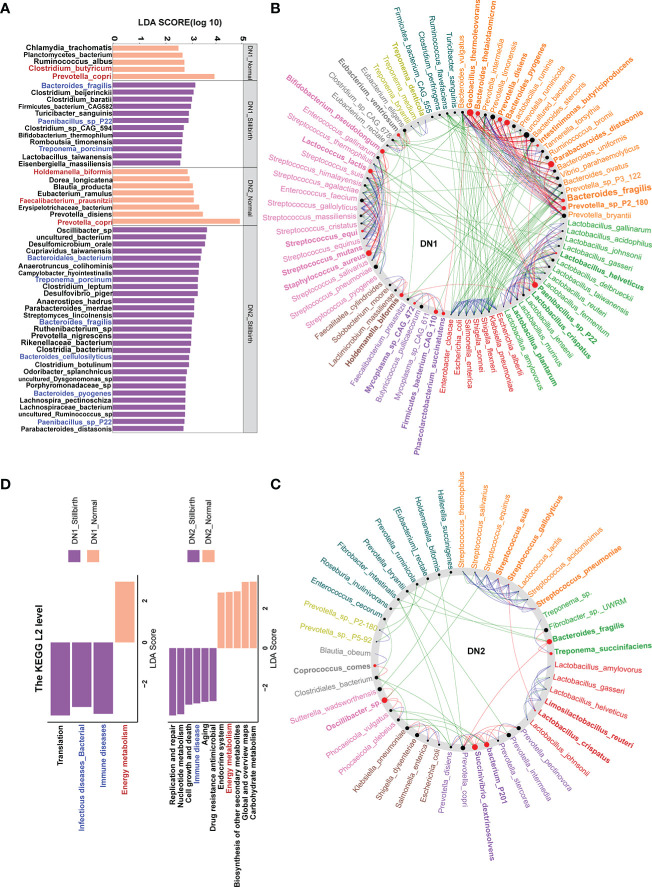
Bacterial species and functional capacities showing differential abundances between stillbirth and normal sow group in the DN1 and DN2 cohort based on metagenomic sequencing data. **(A)** Differential bacterial species between stillbirth and normal sows by LEfSe analysis (LDA score > 2.5 and *P* < 0.05). Blue taxa names represent the bacterial species or genus which were enriched in stillbirth sows from both DN1 and DN2 cohorts, and red taxa names indicate the species or SCFA-producing species which were enriched in normal sows from both DN1 and DN2 cohorts. **(B, C)** Co-occurrence networks of differential bacterial species in the DN1 **(B)** and DN2 **(C)** cohort using the NetShift tool. Nodes size showes the predicated “driver” scores, and red nodes indicate those species identified as important “drivers”. **(D)** Differential KEGG pathways between stillbirth and normal sow group by LEfSe analysis. Blue pathway names represent the diseases-associated pathways which were enriched in stillbirth sows from both DN1 and DN2 cohorts, and the red pathway name represents the pathway enriched in normal sows from both DN1 and DN2 cohort.

To identify bacterial species having different abundances between stillbirth and normal sows, 72 fecal samples from the DN1 and DN2 cohorts were performed metagenomic sequencing analysis (Methods). Among the 20 most abundant species in both cohorts, *Escherichia coli*, *Prevotella copri, Lactobacillus johnsonii* and *lactobacillus reuteri* were predominant (**Figures S3A**, **S3B**). The bacterial species with relative abundance ≥ 0.01% in each cohort were used for association analysis. LEfSe analysis detected 17 and 37 bacterial species showing significantly different abundances between stillbirth and normal sows in the DN1 and DN2 cohort, respectively. (**Figure 4A**, **Supplement Table S4**). Interestingly, three *Bacteroides* species including *Bacteroides fragilis*, *Bacteroides cellulosilyticus* and *Bacteroides pyogenes, Paenibacillus* sp. *P22, Treponema porcinum* were mainly enriched in the stillbirth sow group. Consistent with the results from 16S rRNA gene sequencing, several bacterial species that could produce SCFAs had higher abundance in the gut of normal sows, such as *Clostridium butyricum, Eubacterium ramulus, Faecalibacterium prausnitzii* and *Prevotella copri.* Additionally, some pathogens or opportunistic pathogens had higher abundance in the gut of stillbirth sows, such as *Clostridium baratii* and *Turicibacter sanguinis* in the DN1 cohort*, Desulfomicrobium orale, Cupriavidus taiwanensis, Campylobacter hyointestinalis, Desulfovibrio piger, Ruthenibacterium sp, Prevotella nigrescens, Bacteroides cellulosilyticus, Clostridium botulinum, Bacteroides pyogenes*, and *Parabacteroides distasonis* in the DN2 cohort.

Wilcoxon rank-sum test was further used to confirm the differential species identified by LEfSe analysis. A total of 33 and 26 differential bacterial species were detected in the DN1 and DN2 cohorts, respectively. Impressively, all differential bacterial species detected by the LEfSe analysis were also found by the Wilcoxon rank-sum test in the DN1 cohort (**Figure S4A**, **Supplement Table S4**). Especially, *Prevotella copri* and *Clostridium butyricum* which were enriched in the gut of normal sows showed high significance. Similarly, in the DN2 cohort, the high repeatability was observed in the detection of differential bacterial species by LEfSe analysis and Wilcoxon rank-sum test, such as the enrichments of *Bacteroides fragilis*, *Bacteroides cellulosilyticus*, and *Bacteroides pyogenes* in the gut of stillbirth sows, and the significantly higher abundance of several SCFAs-producing bacterial species in the gut of normal sows, e.g., *Eubacterium ramulus, Faecalibacterium prausnitzii* and *Prevotella copri* (**Figure S4B**, **Supplement Table S4**).

To further document the core “driver” species in the complex micro-ecosystem of sow gut related to giving birth to stillborn piglets, we further constructed co-abundance network using the SparCC and NetShift methods. Impressively, *Bacteroides fragilis*, *Bacteroides pyogenes, Bacteroides thetaiotaoicron* and *Oscillibacter* sp. had a high NESH score as the “driver” species in the gut of stillbirth sows from both DN1 and DN2 cohorts (**Figures 4B, C**). Taken together, we suggested that three *Bacteroides* sp., especially *Bacteroides fragilis* which was detected in both DN1 and DN2 cohorts by both LEfSe and Wilcoxon rank-sum test, and showed the most significant correlation in the DN1 cohort in the LEfSe analysis might be the key bacterial species related to give birth to stillborn piglets in both cohorts, while some SCFAs-producing bacteria, such as *Clostridium butyricum*, *Faecalibacterium prausnitzii*, and *Prevotella copri* might be the key bacterial species in the gut of sows giving birth normally.

We further detected the differential functional pathways of gut microbiome between sows giving birth to stillborn piglets and normal sows in the DN1 and DN2 cohort. A total of 4 and 11 KEGG pathways showing significantly differential abundances were identified in the DN1 and DN2 cohort by LEfSe analysis (**Figure 4D**). Interestingly, the KEGG pathways related to diseases, such as immune diseases and bacterial infectious diseases were significantly enriched in the stillbirth sows in both sow cohorts. Meanwhile, the pathways of energy metabolism and carbohydrate metabolism had more abundance in normal sows. These results suggested that the gut microbiota related to sow giving birth to stillborn piglets might have a potential correlation with the immune response of the host.

**Figure 5:**
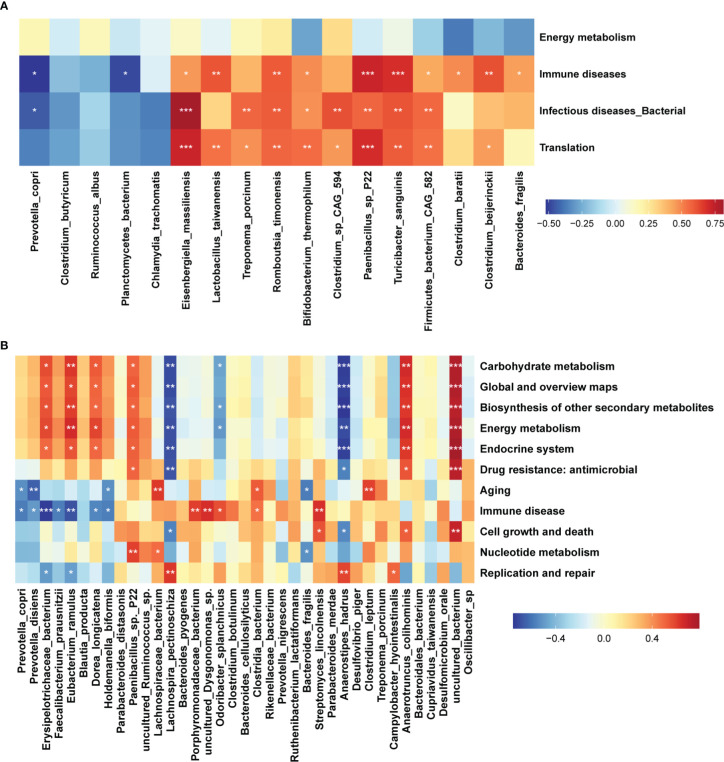
Correlations between differential function pathways and differential bacterial species. Heatmap showing the correlations between differential KEGG pathways of gut microbiome and differential bacterial species. The stars indicate the significance threshold of *P < 0.05, **P < 0.01, and ***P < 0.001 in Spearman’s rank correlation test. **(A)** DN1 cohort, **(B)** DN2 cohort.

We further investigated the correlations between differential bacterial species and differential KEGG pathways in the DN1 and DN2 cohorts by spearman’s correlation analysis (**Figures 5A**, **5B**). In the DN1 cohort, we found that *Bacteroides fragilis*, *Clostridium beijerinckii*, *Clostridium baratii*, *Firmicutes bacterium_CAG_582, Paenibacillus* sp. *P22* and *Turicibacter sanguinis* which were enriched in the stillbirth sow group were positively correlated with the pathways about immune diseases in the DN1 cohort. At the same time, eight of 12 bacteria species which were enriched in the stillbirth sow group were positively correlated with the pathway of infectious diseases, while the *Prevotella copri* which had significantly higher abundance in the normal sow group were negatively correlated with the pathways of immune diseases and infectious diseases. In the DN2 cohort, the *Streptomyces lincolnensis*, *Clostridia bacterium*, *Odoribacter splanchnicus*, *uncultured Dysgonomonas* sp., and *Porphyromonadaceae bacterium* were positively correlated with the pathway of immune disease. Similarly, *Bacteroides fragilis* were positively correlated with the pathway of immune diseases although it did not achieve the statistical significance level (p=0.2 r=0.3). *Prevotella copri*, *Prevotella disiens*, *Erysipelotrichaceae bacterium*, *Faecalibacterium prausnitzii*, *Eubacterium ramulus and Dorea longicatena* which were enriched in normal sows were negatively correlated with immune disease. Interestingly, those bacterial species enriched in the normal sow group, including *Erysipelotrichaceae bacterium*, *Eubacterium ramulus*, and *Dorea longicatena* were positively correlated with the pathways of energy metabolism, carbohydrate metabolism, global and overviews maps, biosynthesis of other secondary metabolites, and endocrine system. Based on these results, we speculated that the sows giving birth to stillborn piglets suffered from abnormal immune response in pregnant stage that might link to the increased abundances of disease-related bacteria and the decreased abundance of bacterial species producing SCFAs.

### The shifts of fecal metabolites in sows giving birth to stillborn piglets

**Figure 6:**
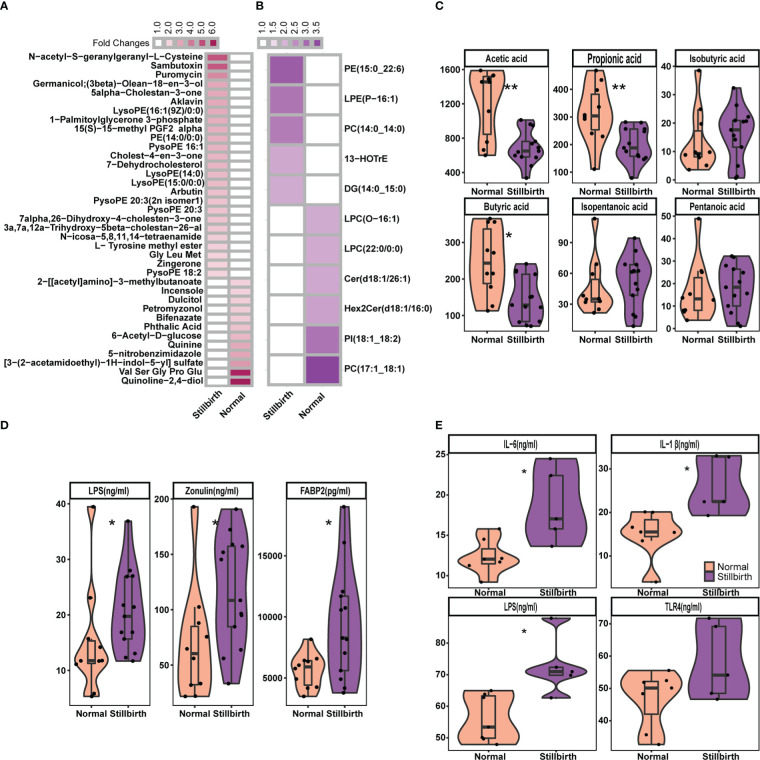
Comparison of the concentration of fecal metabolites, and serum cytokines and biomarkers between stillbirth and normal sow group. The differetial fecal metabolites between stillbirth and normal sow group at the threshold of VIP value > 1.5, wilcox test FDR < 0.05, and fold changes > 2. **(A)** widely targeted metabolome, and **(B)** lipidomic metabolites. **(C)** Comparision of the concentration of fecal SCFAs between stillbirth and normal sow group. **(D)** Comparison of the concentration of serum Lipopolysaccharide (LPS), zonulin and FABP2 in serum samples of DN1 cohort. **(E)** Comparision of the concentration of Lipopolysaccharide (LPS), IL-1β, IL-6 and TLR4 in serum samples from the DN2 cohort. The stars indicate the significance thresholds: * FDR < 0.05 and ** FDR < 0.01 by Wilcoxon rank-sum test.

Many studies have reported that maternal microbiota has an important influence on the fetus development by microbial metabolites translocating to the fetus (19, 67). We first measured the fecal metabolome profile using UPLC-QTOF/MS. A total of 2,185 metabolites were detected from the fecal samples of the DN1 cohort. We performed the Wilcoxon rank-sum test and PLS-DA analysis to identify the differential metabolites between stillbirth and normal sows (**Figure S5A** **)**. At the threshold of FDR < 0.05, fold change > 2 and VIP > 1.5, we detected 37 metabolites showing significantly differential abundance between stillbirth and normal sows (**Figure 6A**). Interestingly, a mass of phosphatidylethanolamine and lysophosphatidylethanolamine, such as LysoPE (16:1(9Z)/0:0), PE (14:0/0:0), PysoPE 16:1, LysoPE 14:0, and LysoPE (15:0/0:0) were enriched in the fecal samples of stillbirth sows (**Figure S6**). In addition, the higher levels of cholesterol including Cholest-4-en-3-one and 7-Dehydrocholesterol, and prototype prostaglandin (15(s)-15-methyl PGF2 alpha) that are the biomarkers of oxidative stress and can cause abortion or the termination of a pregnancy were enriched in fecal samples of the sows giving birth to stillborn piglets (68). However, quinolones related metabolites, e.g., quinolone-2,4-diol and quinine were enriched in fecal samples of the normal sows.

Next, we measured lipidomic metabolites in the same fecal samples described above from the DN1 cohort using LC-ESI-MS/MS. A total of 576 lipid molecules were detected. We also used PLS-DA and Wilcoxon rank-sum test to detect the differential lipidomic metabolites in fecal samples between two sow groups, and found 11 lipid molecules showing significantly differential abundances (**Figure 6B**, **Figure S5B**). Consistently, the phosphatidylethanolamine (PE 15:0_22:6) and lysophosphatidylethanolamine (LPE P-16:1) were also detected to be significantly enriched in fecal samples of the stillbirth sows. However, phosphatidylcholine (PC 17:1_18:1) and lysophosphatidylcholine LPC (O-16:1) and LPC (22:0/0:0) had higher concentration in fecal samples of the normal sows.

Maternal SCFAs are readily transmitted to the offspring, and affect the fetal neural and metabolic systems (69). Compared to sows giving birth to stillborn piglets, normal sows had more SCFAs-producing bacteria in the gut. We assumed whether the concentration of SCFAs in fecal samples should be higher in normal sows. We measured the concentration of fecal SCFAs including acetic acid, propionic acid, isobutyric acid, butyric acid, isopentanoic acid and pentanoic acid in the DN1 cohort using GC-gas chromatograph. As the results, the concentration of acetic acid, propionic acid and butyric acid in fecal samples were significantly higher in the normal sows than that in the sows giving birth to stillborn piglets (**Figure 6C**). However, there were no significant differences in the concentration of of isobutyric acid, isopentanoic acid and pentanoic acid in fecal samples between two sow groups.

### Associations between the changes of gut microbiota and the shifts of fecal metabolites

**Figure 7:**
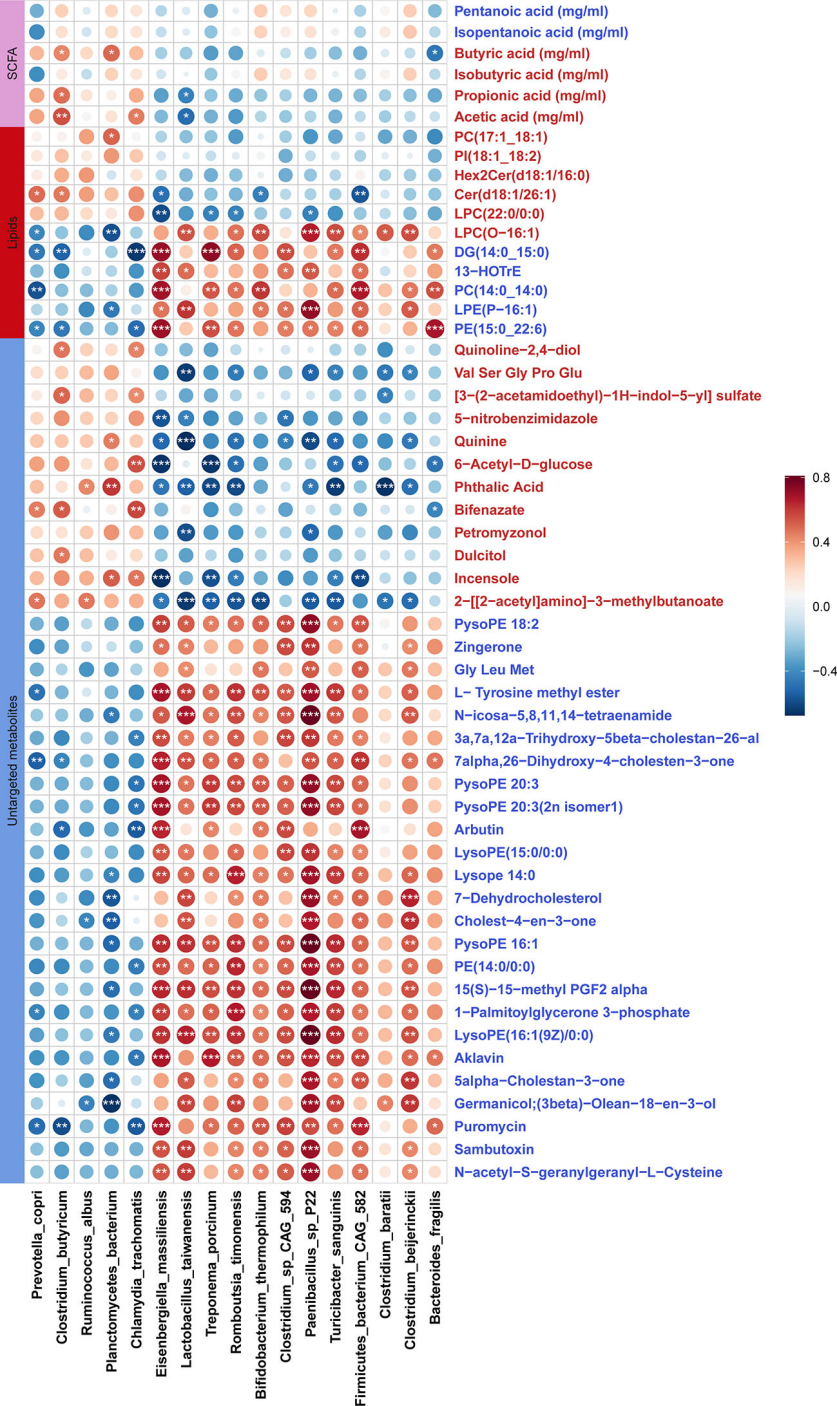
Correlations between differential fecal metabolites and differential bacterial species. The sizes of circles indicate the values of Sperman correlation coefficient. The stars show the significance thresholds: *P < 0.05, **P < 0.01, and ***P < 0.001 by Spearman’s rank correlation test. Blue metabolite names represent the metabolites which were enriched in stillbirth sows, and the red metabolite names show the metabolites which were enriched in normal sows.

We evaluated the correlations between the shifts in fecal metabolites and the changes in the abundances of gut microbial species in the DN1 cohort. In general, the metabolites enriched in fecal samples of the stillbirth sow group were positively correlated with the bacterial species enriched in stillbirth sows, but negatively associated with the bacterial species enriched in normal sows, and vice versa. In details, *Bacteroides fragilis* enriched in the gut of the stillbirth sow group was positively correlated with phosphatidylethanolamine (PE 15:0_22:6) which plays important roles in bacterial adhesion probably *via* affecting LPS biosynthesis (70), but negatively associated with butyric acid (**Figure 7**). *Clostridium butyricm*, a bacterial species producing SCFAs, had a strongly positive correlation with acetic acid, propionic acid and butyric acid. Interestingly, the antibiotics including Aklavin and Puromyclin that were enriched in fecal samples of the stillbirth sows were positively associated with *Bacteroides fragilis*, which might be related to the strong drug resistance of *Bacteroides fragilis (* 71).

### Chronic inflammatory response in sows giving birth to stillborn piglets

Several *Bacteroides* species, especially *Bacteroides fragilis* were enriched in the gut of sows giving birth to stillborn piglets. Alexandrov et al. reported that *Bacteroides fragilis* could produce a pro-inflammatory and neurotoxic LPS (72). We determined and compared the concentration of serum LPS using an ELISA between stillbirth and normal sows in both DN1 and DN2 cohorts. As expected, sows giving birth to stillborn piglets had significantly higher concentration of serum LPS compared to normal sows in both cohorts (**Figures 6D**, **6E**). In addition, we measured the concentration of biomarkers of the gut barrier permeability (zonulin and FABP2) in serum samples from the DN1 cohort. Compared to normal sows, the sows giving birth to stillborn piglets had significantly higher concentration of zonulin and FABP2 in serum, suggesting an increased intestinal barrier permeability (**Figure 6D**). The concentration of pro-inflammatory cytokines IL-6 and IL-1β, and the LPS receptor of TLR4 in serum were also measured by ELISA assay in the DN2 cohort. The levels of IL-6 (*P* = 0.02) and IL-1β (*P* = 0.01) were significantly higher in the serum of stillbirth sows than that in normal sows (**Figure 6E**). The concentration of TLR4 in the serum was also higher in the stillbirth sow group although this did not achieve the statistical significance level.

**Figure 8:**
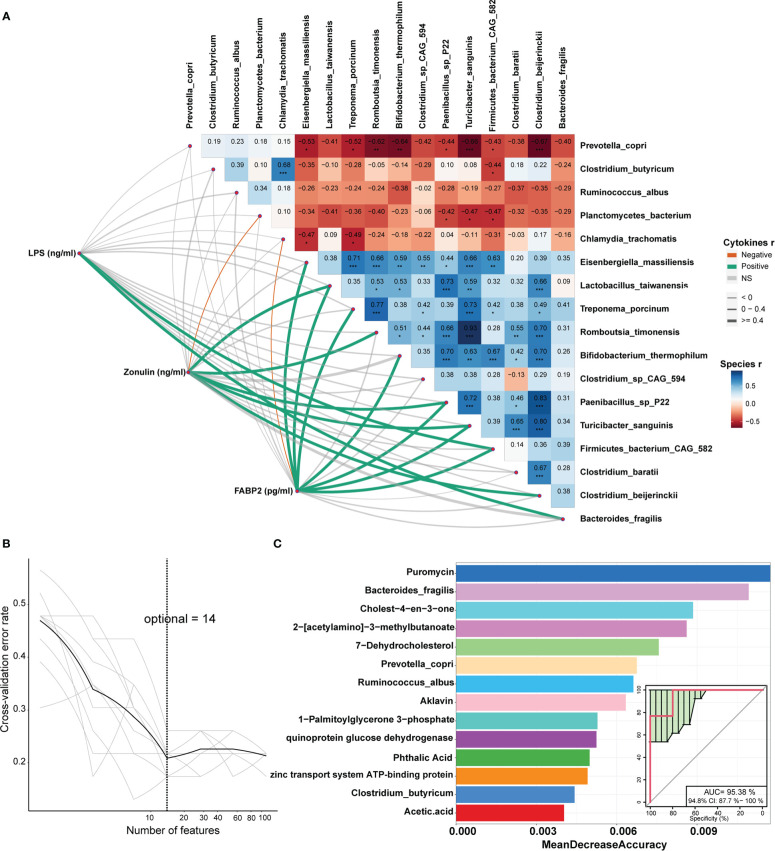
Correlations between cytokines and differential bacterial species and identification of the biomarkers that could be used to classify the sows giving birth to stillborn piglets by Random Forest. **(A)** The values on the heatmap represent the Spearman correlation coefficient between bacterial species which were enriched in each of two sow groups. *, *P* < 0.05, **, *P* < 0.01, and ***, *P* < 0.001 obtained in the Spearman’s rank correlation test. The lines represent the Spearman correlation between cytokines and bacterial species, the green lines indicate a positive correlation, the orange lines show a negative correlation, and the grey lines indicate the correlation that did not achieve significance level. **(B)** The tenfold cross-validation by Random Forest model. **(C)** The 14 features that could discriminate the stillbirth and normal sows.

We further evaluated the correlations between the shifts in the concentrations of serum inflammation cytokines and LPS, and the changes in the abundances of gut microbial species in the DN1 cohort. Serum LPS abundance showed robust correlation with *Bacterial fragilis* that was enriched in stillbirth sows. Moreover, FABP2 and zonulin were positively correlated with most of the stillbirth-associated bacterial species (**Figure 8A**). Taken together, these results suggested that the changes in the gut microbiota of sows, e.g., an excess abundance of *Bacteroides fragilis* produced high concentration of LPS which impaired intestinal integrity, activated sow pro-inflammatory response, induced the production of inflammatory cytokines, and finally resulted in the death of relatively weak piglets in a farrow.

### Identifying the biomarkers predicting the sows giving birth to stillborn piglets

In order to identify the potential biomarkers that could be used to predict the sows giving birth to stillborn piglets, we constructed random-forest classifiers to discriminate stillbirth and normal sows based on 112 differential features including bacterial species (n = 17), KO (n = 36), metabolites (n = 56) and inflammation cytokines (n = 3). A tenfold cross-validation method was used to identify the features with the best discriminatory power. The result showed that 14 features could distinguish stillbirth and normal sows well (**Figure 8B**). Interestingly, among these 14 features, puromycin had the best discriminatory power (top 1). As the antibiotic, it was significantly correlated with the abundance of *Bacteroides fragilis*. Moreover, the species *Bacteroides fragilis, Prevotella copri, Ruminoccus albus* and *Clostridium butyricum* were also included in the list of these 14 biomarkers. The prediction model showed high diagnostic power with the area under the curve of AUC 95.38% (**Figure 8C**), suggesting that these 14 features could be used as biomarkers for predicting the occurrence of stillbirth sows.

## Discussion

Sows giving birth to stillborn piglets bring a big loss to pig production industry. The reasons causing stillborn piglets are particularly complicated (1, 10, 73). Here, to our knowledge, for the first time, we showed that sow gut microbes in late trimester of pregnancy might play an important role in the occurrence of stillborn piglets in three experimental sow cohorts by integrating multi-omics data.

We did not find any CAGs showing significant difference in abundance between normal and stillborn groups in either DN1 or DN2 cohort although some of CAGs showed the tendency to significance. This should be due to: 1) Although we identified some bacterial taxa or species showing significantly different abundance between normal and stillborn groups, the shifts in the abundances of these bacterial taxa did not significantly change the compositions and interactions of bacterial taxa between normal and stillborn groups. We did not observe the significant difference in the overall diversity of gut microbiota between two groups (both α- and β-diversity did not show significant differences between normal and stillborn groups) (**Figure S1**). 2) The sample sizes were relatively small for both DN1 and DN2 cohorts, so it only showed the tendency to significance.

Several *Bacteroides* spp., such as *Bacteroides fragilis, Bacteroides pyogenes*, and *Bacteroides thetaiotaomicron*, and *Treponema porcinum* were detected to be enriched in the gut of sows giving birth to stillborn piglets by metagenomic sequencing analysis. Moreover, several pathogen or opportunistic pathogens including *Clostridium baratii, Desulfomicrobium orale, Cupriavidus taiwanensis, Campylobacter hyointestinalis, Desulfovibrio piger, Bacterioides cellulosilyticus, Odoribacter splanchnicus*, and *Porphyromonadaceae bacterium* (74–79), and LPS-producing species, e.g., *Bacteroides fragilis* (72) had the higher abundances in the stillbirth sows. Particularly, *Bacteroides fragilis* was enriched in stillbirth sows by both LEfSe analysis and Wilcoxon rank-sum test and had the highest LDA score in the DN1 cohort in LEfSe analysis. Considering the high abundance of *Bacteroides fragilis* in the gut of stillbirth sows, and particularly, that *Bacteroides fragilis* was recognized as a core driver species in the gut microbial community of stillbirth sows by NetShift network analysis in both DN1 and DN2 cohorts, we speculated that the excessive enrichment of *Bacteroides fragilis* in the gut could result in stillborn piglets. *Bacteroides fragilis* is one of the bacterial species having outstanding ability to produce polysaccharides (80). It is also one of common anaerobic bacteria causing the infection, and the infection rate is high if the integrity of intestinal mucosa is impaired (77, 81). We indeed detected that serum FABP2 and zonulin, the biomarkers of intestinal permeability, had higher concentration in sows giving birth to stillborn piglets than that in normal sows, indicating an increased intestinal barrier permeability in sows with stillbirth piglets. Seungbum et al. also showed that zonulin and FABP2 were correlated with serum LPS and altered gut microbiota that were related to intestinal inflammation (82). *Bacteroides fragilis* is an obligate anaerobic gram-negative bacterium whose outer membrane is a highly asymmetric bilayer that contains phospholipids in the inner leaflet and LPS in the outer leaflet (83). Extensive studies have confirmed the relationship of LPS with host inflammatory response (84, 85). Toyama et al. reported that LPS could cause fetal death or abortion in animals (86). We detected that the level of LPS was higher in the stillbirth sows and positively correlated with the abundance of *Bacteroides fragilis.* In addition, fecal metabolome and lipidomics analysis identified that phosphatidylethanolamine (PE) and related lipids (such as lysoPE and pysoPE) were enriched in the stillbirth sow group. However, the serum levels of phosphatidylcholine (PC) and lysophosphatidylcholine (LPC) that play a role in the regulation of cell immunes response, had the higher concentration in normal sows. LPC can inhibit TLR-mediated intracellular responses, and ultimately induced an anti-inflammatory phenotype (87). PE is the principal phospholipid in bacteria. In Gram-negative bacteria, e.g., *Escherichia coli*, PE takes about 70–80% of the total membrane lipids (70). Yu et al. also reported that PE had an important influence on bacterial adhesion probably *via* affecting LPS biosynthesis (70). The abundance of *Bacteroides fragilis* showed the most significant correlation with the concentration of PE. These results further suggested that more *Bacteroides fragilis* adhering to the gut of sows giving birth to stillborn piglets produced higher concentration of LPS that increased intestinal barrier permeability and chronic inflammation response. It has widely known that *Bacteroides fragilis* has two different strains, non-toxigenic (NTBF) and enterotoxigenic (ETBF). *Bacteroides fragilis* toxin (BFT) is the only well-studied virulence factor specific to ETBF (88). We blasted the metagenomic sequencing reads into *bft* genes, but didn’t detect *bft* gene sequence in the samples from experimental pigs (data not shown), indicating that the *Bacteroides fragilis* should be non-toxigenic subtype in tested samples. Kordahi et al. reported that the non-toxigenic *Bacteroides fragilis* from patients were enriched the genes involving in the LPS biosynthesis, could activate NF-κB through TLR4, and induced a pro-inflammatory response (89). Toll-like receptors (TLRs) are responsible for the recognition of LPS and play a central role in the initiation of innate immune responses (84, 90). We presumed that non-toxigenic *Bacteroides fragilis* enriched in the sows giving birth to stillborn piglets might activate the host immune system and cause inflammation response. As we expected, the serum levels of TLR4, IL-6 and IL-1β had higher concentration in the stillbirth sow group. Previous study also indicated that LPS can medicate fetal death by inducing the substantial increase in decidua COX-2 (91). Taken together, LPS produced by stillborn-associated bacterial taxa, e.g., non-toxigenic *Bacteroides fragilis* strains might impair intestinal integrity, activate sow pro-inflammatory response, induce the production of inflammatory cytokines, and finally result in the death of relatively weak piglets in a farrow.

Several SCFA-producing bacteria, such as *Clostridium butyricum, Eubacterium ramulus, Faecalibacterium prausnitzii* and *Prevotella copri* were enriched in the gut of normal sows. SCFAs not only provide energy for host cells, but also serve as signal molecules between gut microbiota and intestinal organs, and can inhibit intestinal inflammation response (92–94). Metagenomic sequencing analysis showed that the pathway of energy metabolism was enriched in normal sows, indicating that SCFAs-producing microbes should benefit pregnancy sows by providing SCFAs that provided sows energy and also inhibited host inflammation response. This finally improved the development of the fetal. SCFAs regulate host energy homeostasis *via* GPR41 and GPR43 in the sympathetic nervous system, adipose, pancreas, intestine and embryo tissues, and play anti-inflammatory roles (20, 95). In pregnant mice, acetate produced by gut microbiota can permeate the placenta and attenuate postnatal allergic responses in the offspring (69). Maternal gut microbiota could affect maternal and placental metabolome (i.e. acetate, formate and carnitine), and then promote placental morphogenesis, nutrient transport and fetal growth in mice (18). We indeed observed that normal sows had significantly higher concentration of SCFAs in feces than sows giving birth to stillborn piglets. *Clostridium butyricum* and *Faecalibaverium prausnitzii* were significantly associated with the levels of feces SCFAs. Maternal carriage of *Prevotella copri* during pregnancy may promote the development of fetal immune tolerance (96). Different *Prevotella copri* strains have shown distinct functional capacities depending on the diets (97).

## Conclusion

Overall, pregnant sows with higher abundances of bacterial species from *Bacteroides* (especially *Bacteroides fragilis*) and *Treponema porcinum* in the gut had a relatively higher risk of giving birth to stillborn piglets. LPS produced by stillbirth-associated bacterial species might impair intestinal barrier, activate the host immune response, and induce the production of inflammatory cytokines, and finally result in the death of relatively weak piglets (stillborn piglets) in a farrow of experimental sows. However, some SCFAs-producing bacterial species provided SCFAs that not only provided the host more energy, but also inhibited host inflammation response, and should benefit for normal development of fetus. The results from this study gave the insights into how maternal gut microbiome affects the development of fetus, and provided the basic knowledges to decrease the occurrence of sows giving birth to stillborn piglets through regulating the sow gut microbiota to benefit for the pig production.

## Data availability statement

All 16S rRNA gene sequencing data and metagenomic sequencing data were submitted to the China National Center for Bioinformation database with the accession number CRA008769 and CRA008770.

## Ethics statement

The animal study was reviewed and approved by the Ministry of Agriculture and Rural Affairs (MARA) of China and the Animal Care and Use Committee (ACUC) in Jiangxi Agricultural University (No. JXAU2011-006).

## Author contributions

LH designed the research and revised the manuscript. CC designed the research, wrote and revised the manuscript. ZC and HY performed experiments, analyzed data and wrote the manuscript. HF assisted in collecting samples. LW, ML and QL assisted in extracting the DNA from fecal samples. HJ assisted in performing serum cytokines experiments of ELISA, YW and SX assisted in performing metabolomic experiments of LC-MS, MZ and XS assisted in quantitatively analyzing the concentration of short-chain fatty acids. All authors contributed to the article and approved the submitted version.

## Supplementary material

Supplementary Figure 1 The factors influencing gut microbial composition and the comparison of α-diversity of gut microbial compositions between stillbirth and normal sows based on 16S rRNA gene sequencing data. **(A)** The CCA analysis was used to evaluate the effects of the factors including population, number of stillbirths, parity and maternal infanticide on the gut microbial composition based on 16S rRNA gene sequencing data. **(B)** Comparison of the α-diversity of gut microbiome between stillbirth and normal sow group using richness, ACE, chao, Shannon and Simpson indices. Boxplots showing the difference of each index between stillbirth and normal sow group in the F6, DN1, D2 cohort by Wilcoxon rank-sum test, and a false discovery rate (FDR) < 0.05 was set as the significance threshold.

Supplementary Figure 2 OTUs significantly enriched in stillbirth sows and normal sows based on 16S rRNA gene sequencing data by Wilcoxon rank-sum test (central log-ratio transformation). The volcano plots show the differential OTUs with corrected P value (FDR) < 0.05 and log2 FC > 0. The circles represent the OTUs which were enriched in the stillbirth group and the triangles indicate the OTUs which were enriched in the normal group. Purple circles show the OTUs belonging to Clostridiales, orange triangles indicate the OTUs belonging to Bacteroidales, and the grey ones indicate the differential OTUs annotated to other taxonomy. **(A)** F6 cohort, **(B)** DN1 cohort, **(C)** DN2 cohort.

Supplementary Figure 3 Co-abundance network analysis based on 16S rRNA gene sequencing data in the F6 cohort. **(A)** Co-abundance network analysis of OTUs in the F6 cohort. **(B)** Differential abundance of CAGs in the F6 cohort between stillbirth and normal sow group by Wilcoxon rank-sum test.

Supplementary Figure 4 The bacterial species whose abundances was listed in the top 20 in the DN1 and DN2 cohort based on metagenomic sequencing data and differential bacterial species identified by Wilcoxon rank-sum test (central log-ratio transformation). Stackplot with the same color repsents the same bacterial species in the DN1 **(A)** and DN2 **(B)** cohort. The volcano plots show the bacterial species which were differentially enriched in stillbirth sows and normal sows in the DN1 **(C)** and DN2 **(D)** cohorts with corrected P value (FDR) > 0.05 and log2 Fold Change > 0, the purple circles represent the bacterial species which were enriched in stillbirth sows and the orange triangles indicate the bacerial species that were enriched in normal sows.

Supplementary Figure 5 The distribution of fecal metabolite profiles between stillbirth and normal sows. The Partial Least Squares Discriminant Analysis (PLS-DA) showed that both untargeted metabolites **(A)** and lipid molecules **(B)** profiles showed different distribution between the stillbirth and the normal sow group from DN1 and DN2 cohort.

Supplementary Figure 6 Comparison of the concentration of PGF2 alpha, PE and lysoPE between stillbirth and normal sow group in the DN1 cohort by Wilcox sum-rank test. False discovery rate (FDR) < 0.05 was set as the significance threshold.
