# Supplementary figures and images for: Gut bacterial species in late trimester of pregnant sows influence the occurrence of stillborn piglet through pro-inflammation response
Source: Front Immunol. 2023 Jan 18;13:1101130. doi: 10.3389/fimmu.2022.1101130 (PMC9890068; doi:10.3389/fimmu.2022.1101130)

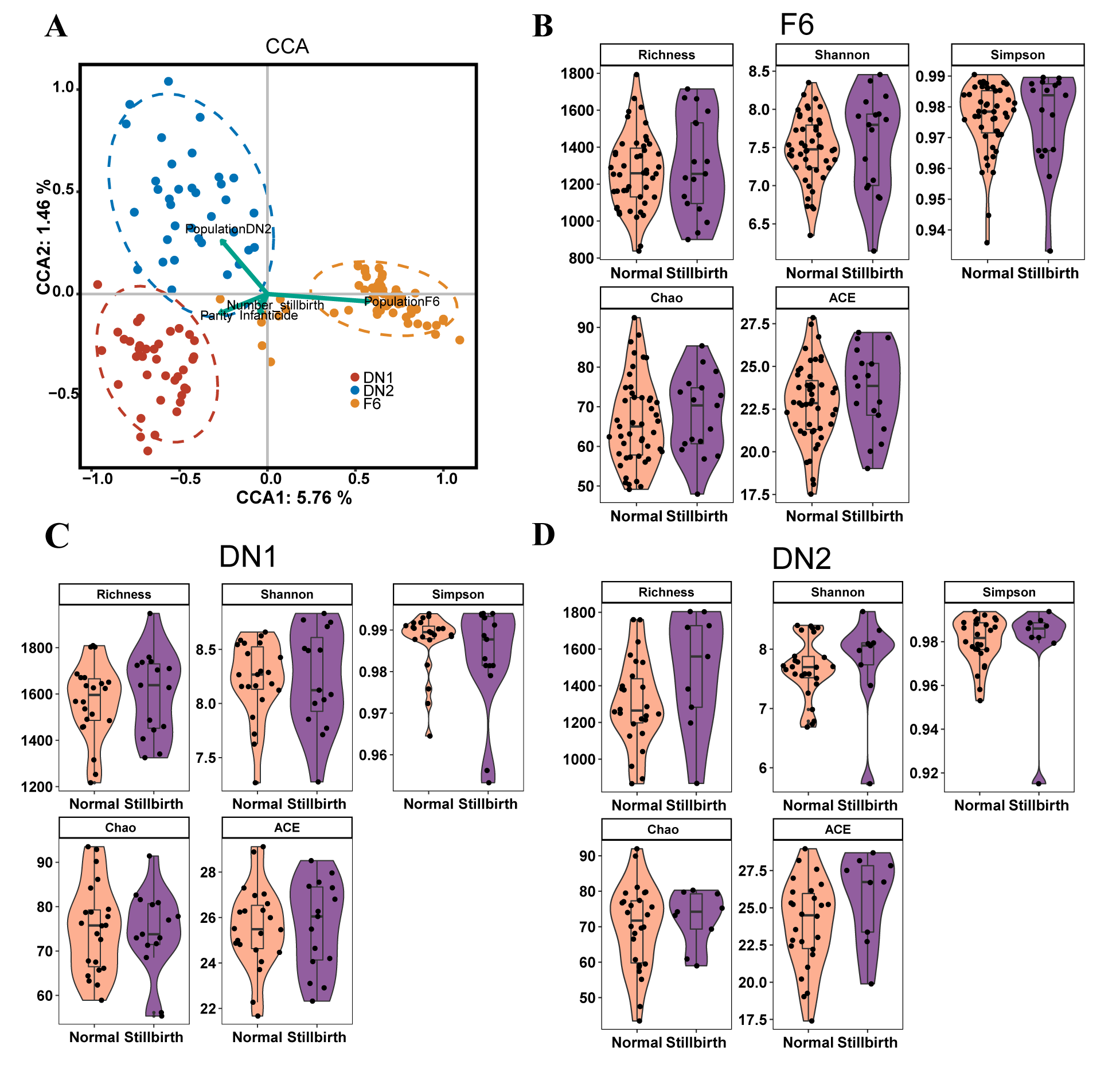

Supplement: Supplementary Figure 1 — The factors influencing gut microbial composition and the comparison of α-diversity of gut microbial compositions between stillbirth and normal sows based on 16S rRNA gene sequencing data. (A) The CCA analysis was used to evaluate the effects of the factors including population, number of stillbirths, parity and maternal infanticide on the gut microbial composition based on 16S rRNA gene sequencing data. (B) Comparison of the α-diversity of gut microbiome between stillbirth and normal sow group using richness, ACE, chao, Shannon and Simpson indices. Boxplots showing the difference of each index between stillbirth and normal sow group in the F6, DN1, D2 cohort by Wilcoxon rank-sum test, and a false discovery rate (FDR) < 0.05 was set as the significance threshold. [file Image_1.tif]

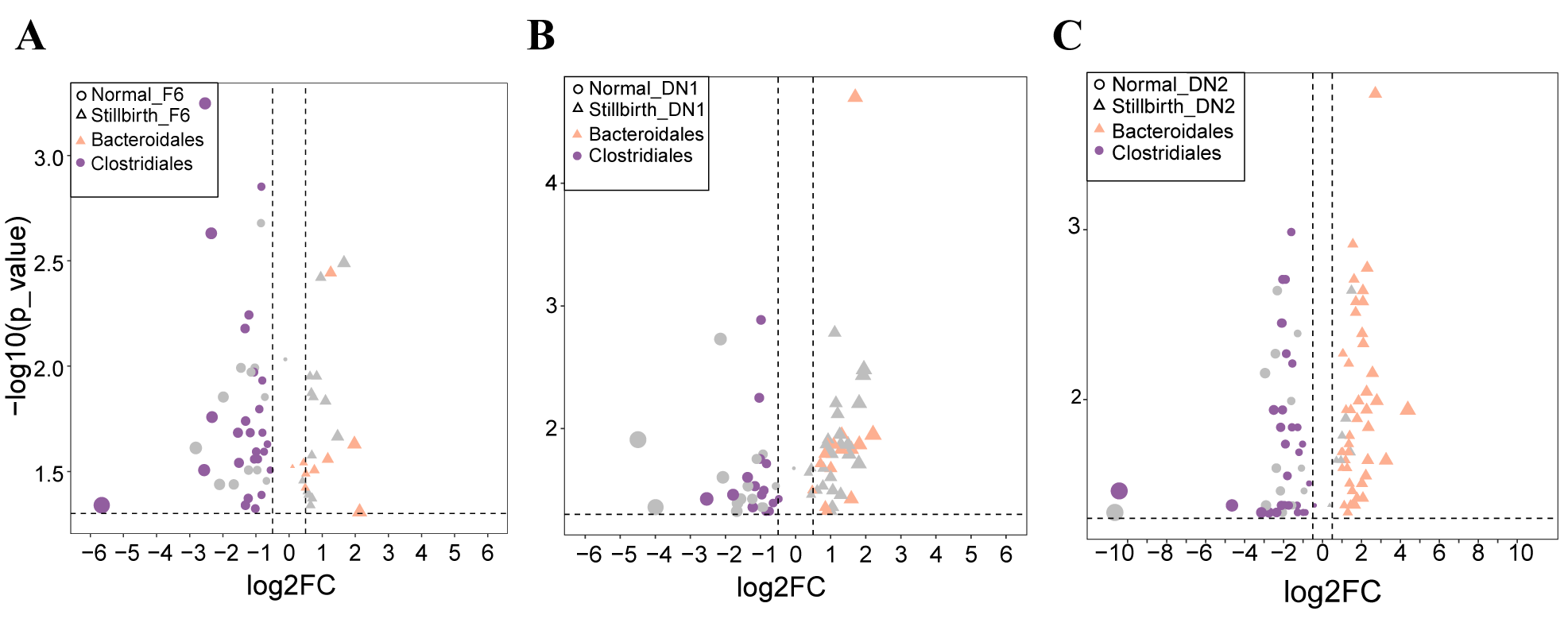

Supplement: Supplementary Figure 2 — OTUs significantly enriched in stillbirth sows and normal sows based on 16S rRNA gene sequencing data by Wilcoxon rank-sum test (central log-ratio transformation). The volcano plots show the differential OTUs with corrected P value (FDR) < 0.05 and log2 FC > 0. The circles represent the OTUs which were enriched in the stillbirth group and the triangles indicate the OTUs which were enriched in the normal group. Purple circles show the OTUs belonging to Clostridiales, orange triangles indicate the OTUs belonging to Bacteroidales, and the grey ones indicate the differential OTUs annotated to other taxonomy. (A) F6 cohort, (B) DN1 cohort, (C) DN2 cohort. [file Image_2.tif]

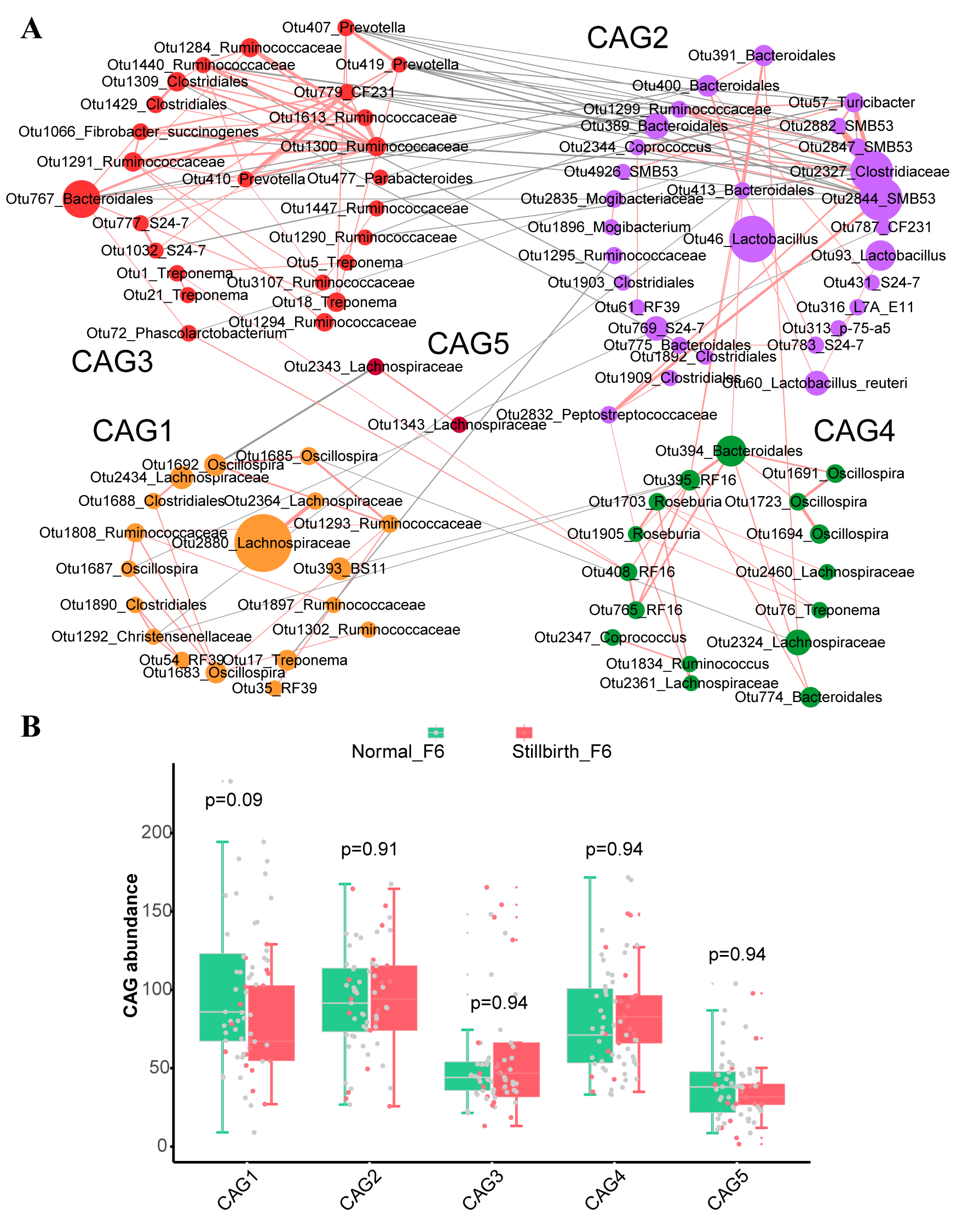

Supplement: Supplementary Figure 3 — Co-abundance network analysis based on 16S rRNA gene sequencing data in the F6 cohort. (A) Co-abundance network analysis of OTUs in the F6 cohort. (B) Differential abundance of CAGs in the F6 cohort between stillbirth and normal sow group by Wilcoxon rank-sum test. [file Image_3.tif]

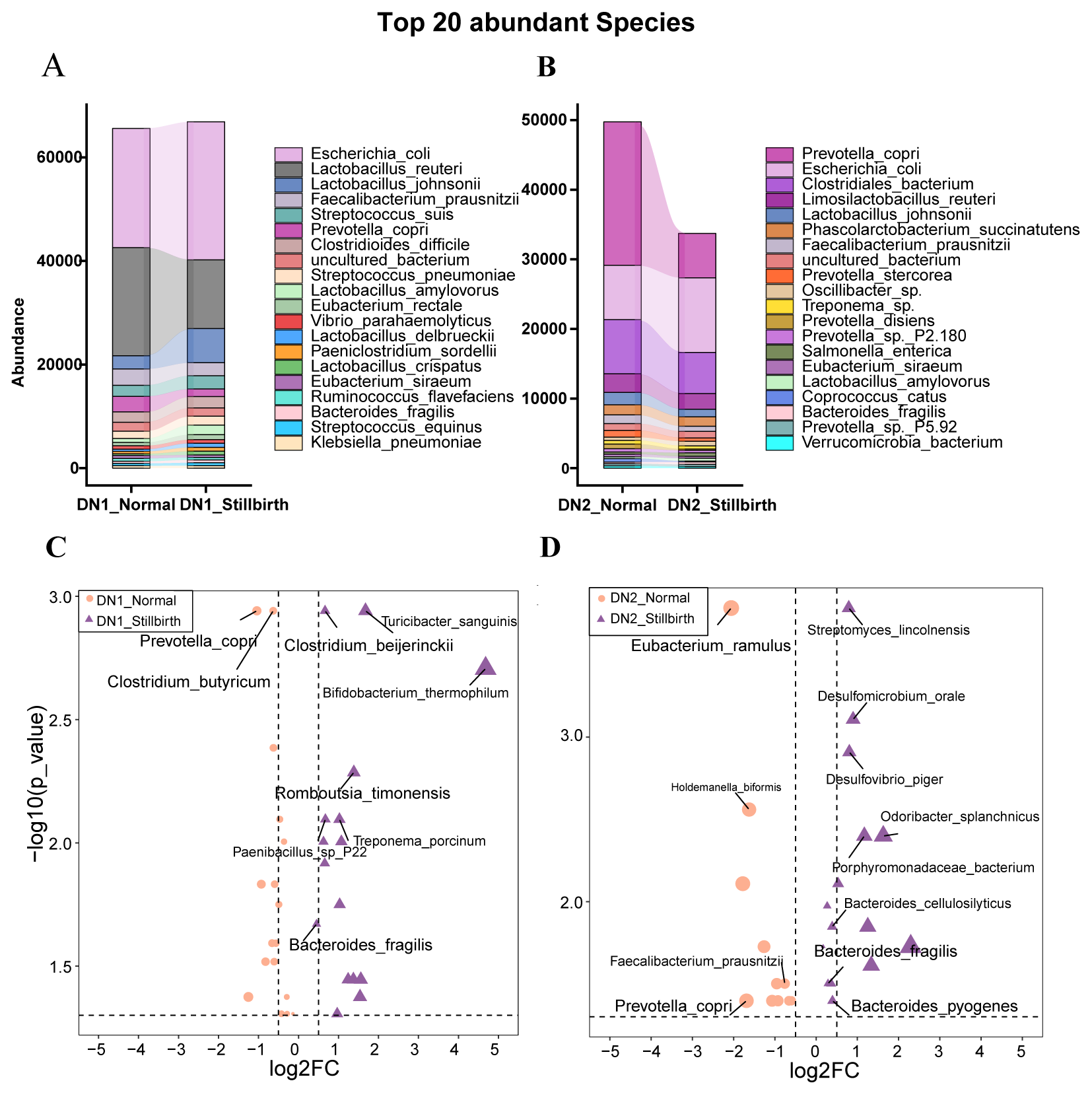

Supplement: Supplementary Figure 4 — The bacterial species whose abundances was listed in the top 20 in the DN1 and DN2 cohort based on metagenomic sequencing data and differential bacterial species identified by Wilcoxon rank-sum test (central log-ratio transformation). Stackplot with the same color repsents the same bacterial species in the DN1 (A) and DN2 (B) cohort. The volcano plots show the bacterial species which were differentially enriched in stillbirth sows and normal sows in the DN1 (C) and DN2 (D) cohorts with corrected P value (FDR) > 0.05 and log2 Fold Change > 0, the purple circles represent the bacterial species which were enriched in stillbirth sows and the orange triangles indicate the bacerial species that were enriched in normal sows. [file Image_4.tif]

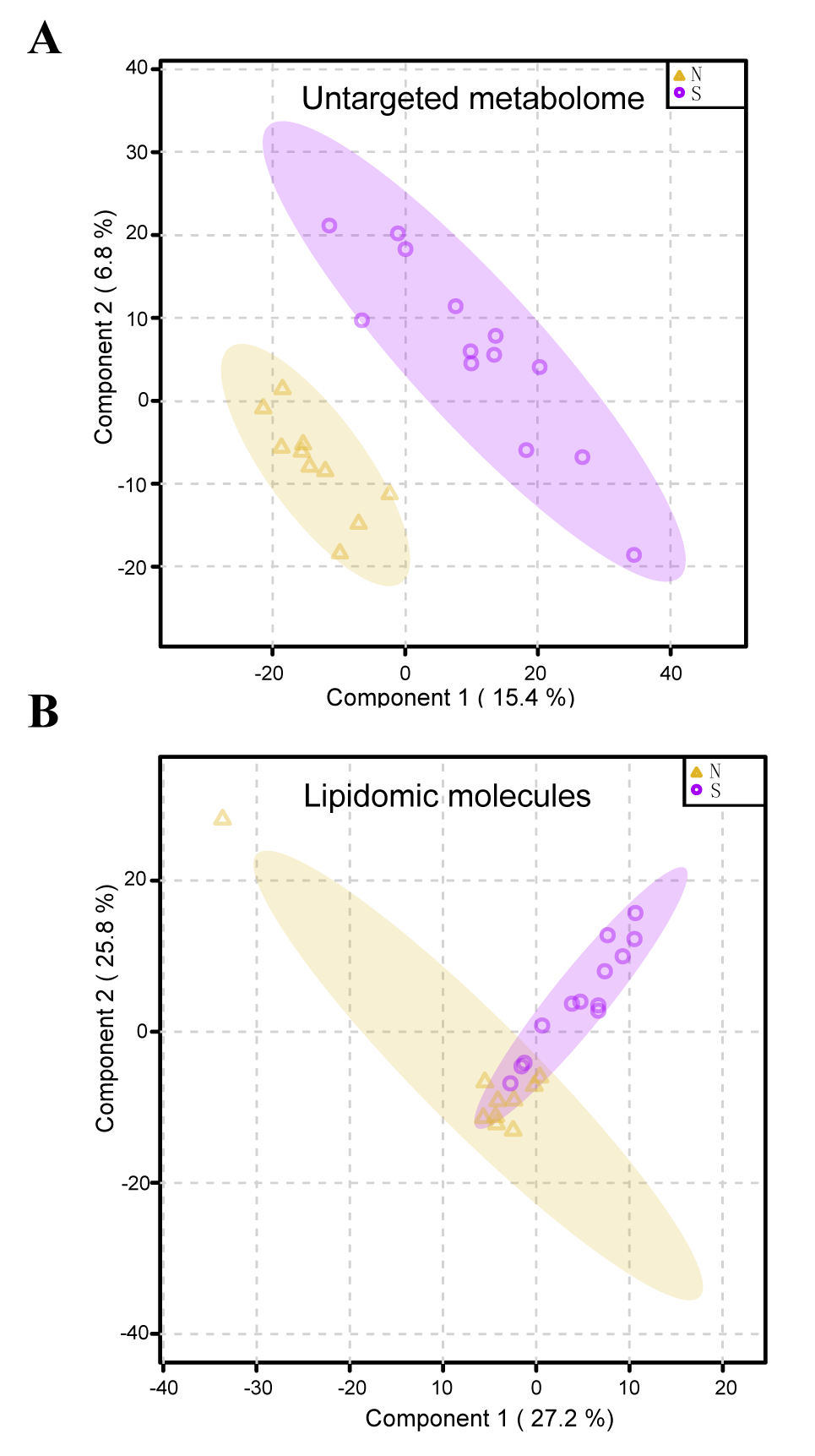

Supplement: Supplementary Figure 5 — The distribution of fecal metabolite profiles between stillbirth and normal sows. The Partial Least Squares Discriminant Analysis (PLS-DA) showed that both untargeted metabolites (A) and lipid molecules (B) profiles showed different distribution between the stillbirth and the normal sow group from DN1 and DN2 cohort. [file Image_5.tif]

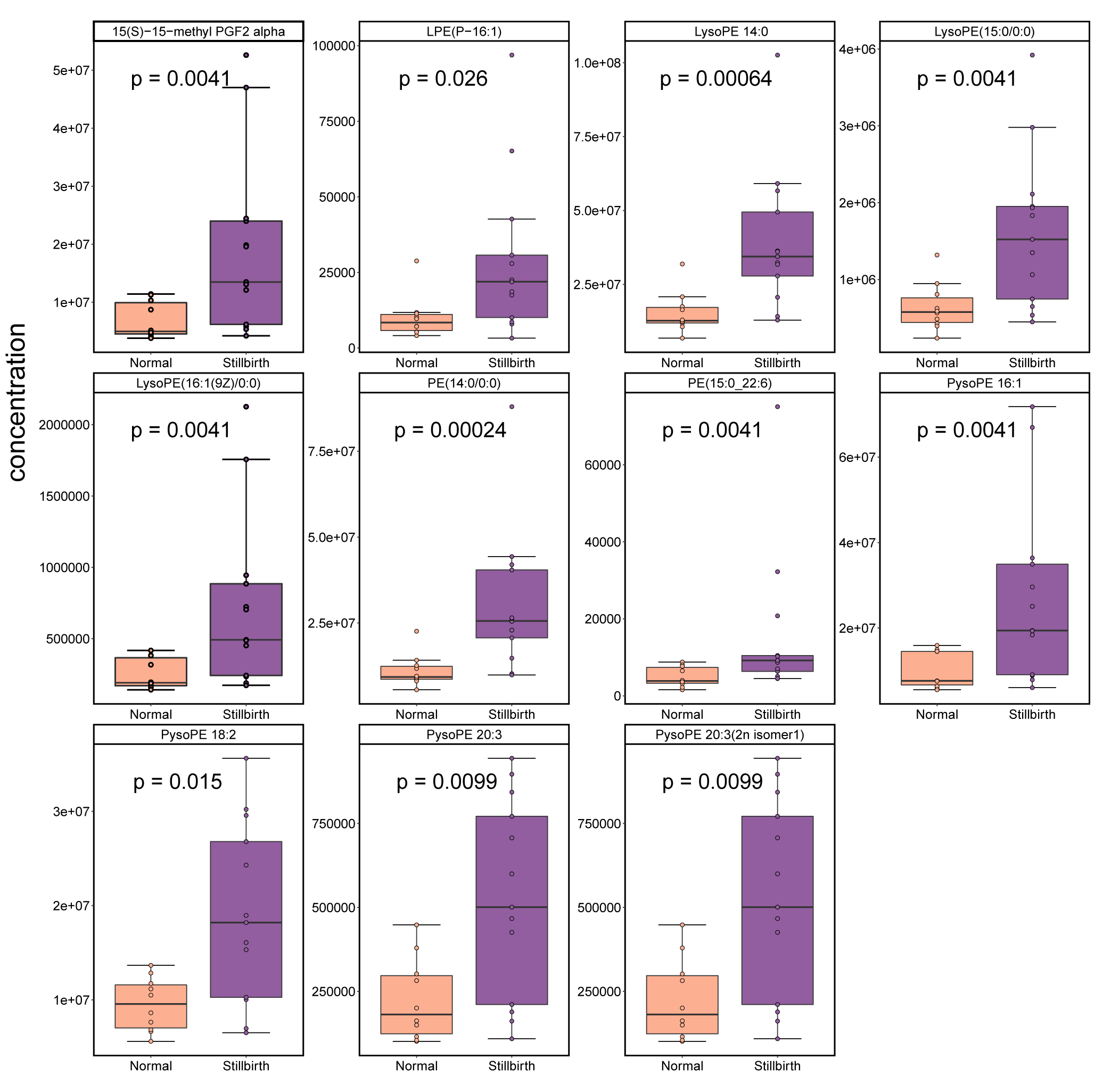

Supplement: Supplementary Figure 6 — Comparison of the concentration of PGF2 alpha, PE and lysoPE between stillbirth and normal sow group in the DN1 cohort by Wilcox sum-rank test. False discovery rate (FDR) < 0.05 was set as the significance threshold. [file Image_6.tif]
